# The protease bromelain breaks oral tolerance and promotes sensitization of mice in a adjuvant-free murine model of pineapple (*A.comosus*) anaphylaxis

**DOI:** 10.1186/1939-4551-8-S1-A156

**Published:** 2015-04-08

**Authors:** Fernanda Bruni, Jorge Kalil, Fernanda Arantes Costa, Fabio FM Castro

**Affiliations:** 1 School of Medicine of University of Sao Paulo, Brazil; 2 University of São Paulo, Brazil; 3 Heart Institute (InCor), Brazil; 4 Asbai, Brazil

## Background

Allergies represent a socioeconomic problem that has affected increasing world population. A range of respiratory allergens are themselves proteases or are associated with proteolytic activity. Bromelain is a mixture of cysteine proteases present in the leaves and fruit *Ananas comosus*, with several pharmacological applications and is widely used in the food industry. Cases of occupational allergy are described in the literature. We propose investigate the contribution of Bromelain in anaphylaxis induced by *Ananas comosus* in a murine model.

## Methods

Female Balb/c mice were maintained fasting for 7 hours and then sensitized with active Bromelain through intragastric route, once a week per 8 consecutive weeks. The body weight and blood sample are collected in 0, 21, 42 and 56 days. In day 56, animals were challenge by oral route with active Bromelain or fresh *A.comosus* (pineapple) extract . Early signals, rectal temperature and respiratory parameters (by Whole-body plethysmograph) were measured by up to 45 minutes.IgG1 and IgG2a specific-antibodies were quantified by ELISA and anaphylatic antibodies title was obtain by Active Anaphylaxis Cutaneous (ACA). Histological methods were realized for measure infiltrate in stomach and small intestine.

## Results

Bromelain group mice were underweight when compared to the saline group. The oral challenge (day 56) promoted a drop of the body temperature when mice were challenge to both Bromelain and fresh pineapple extract. Decreases in expiratory time and relation time were induced by oral challenge with protease. Specific IgG1 and IgG2a were detected in the serum of sensitized mice. Antibodies produced in response to Bromelain promoted a positive and high title of anaphylactic antibodies (1/40) by ACA for fresh *A.comosus* extract and a smaller title for Bromelain. In addition, after 24 hour to oral challenge, sensitized mice presented infiltrate inflammatory cells in stomach and small intestine.

## Conclusions

Using the mouse model of food allergy previously developed by us, we describe here the ability of majority protease Ananas, Bromelain, in breakdown oral tolerance and functions as Th2 adjuvant, sensitizing mice to respond positively after challenge to both fresh *A. comosus* extract and Bromelain.

## Financial support

FAPESP and Institute for Investigation in Immunology (iii)–INCT-CNPq.

